# AGE DIFFERENCES AMONG WITHIN-PERSON INDICATORS OF STRESS AND DEPRESSION

**DOI:** 10.1093/geroni/igad104.0078

**Published:** 2023-12-21

**Authors:** Jessica Blaxton, Cindy Bergeman

**Affiliations:** Metropolitan State University, Saint Paul, Minnesota, United States; University of Notre Dame, Notre Dame, Indiana, United States

## Abstract

Although older adults tend to regulate their emotions and resist stress better than midlife or younger adults (Charles & Carstensen, 2007), research suggests that when older adults do negatively react to stress they may have more difficulty recovering (Charles, 2010). Research reveals that various indicators of stress relate to greater depression – even suggesting that stress causes the depression (van Praag, 2004). Individual perceived stress (PS) as well as perceived stress reactivity (PSR) influence how stress impacts well-being (Folkman et al., 1986). Thus, we examined age differences in the within-person relationships among PS, PSR, and depression, and potential causal determinants of depression with a longitudinal mediation model. We used data from 572 participants in the Notre Dame Study of Health & Well-being (M(age) = 59.77; sd(age) = 14.22) who completed two to four waves of yearly assessments. Sequentially built multilevel models, in which year was nested within person, illustrated that only midlife adults experience an exacerbated effect of within-person fluctuations in PSR on the relationship between within-person PS and depressive levels (gamma41 = -0.004, p < .01). The longitudinal mediation model revealed that PSR at Time 2 mediated the relationship between PS at Time 1 and Depression at Time 3. Findings suggest that older adults illustrate successful emotion regulation strategies at the yearly level --resisting the negative ramifications of years of greater PS and PSR, whereas midlife adults who experience years of greater PSR would particularly benefit from stress management interventions and monitoring of depressive levels.

